# N-Acetylcysteine Attenuates Diabetic Myocardial Ischemia Reperfusion Injury through Inhibiting Excessive Autophagy

**DOI:** 10.1155/2017/9257291

**Published:** 2017-02-06

**Authors:** Sheng Wang, Chunyan Wang, Fuxia Yan, Tingting Wang, Yi He, Haobo Li, Zhengyuan Xia, Zhongjun Zhang

**Affiliations:** ^1^Department of Anesthesiology, Guangdong Cardiovascular Institute, Guangdong General Hospital, Guangdong Academy of Medical Science, Guangdong, China; ^2^Department of Anesthesiology, Shenzhen People's Hospital, Shenzhen Anesthesiology Engineering Center, The Second Clinical Medical College, Jinan University, Shenzhen, China; ^3^Department of Anesthesiology, Fuwai Hospital, National Center for Cardiovascular Diseases, Chinese Academy of Medical Sciences and Peking Union Medical College, Beijing, China; ^4^Department of Anesthesiology, The University of Hong Kong, Pokfulam, Hong Kong

## Abstract

*Background*. Excessive autophagy is a major mechanism of myocardial ischemia reperfusion injury (I/RI) in diabetes with enhanced oxidative stress. Antioxidant N-acetylcysteine (NAC) reduces myocardial I/RI. It is unknown if inhibition of autophagy may represent a mechanism whereby NAC confers cardioprotection in diabetes.* Methods and Results*. Diabetes was induced in Sprague-Dawley rats with streptozotocin and they were treated without or with NAC (1.5 g/kg/day) for four weeks before being subjected to 30-minute coronary occlusion and 2-hour reperfusion. The results showed that cardiac levels of 15-F2t-Isoprostane were increased and that autophagy was evidenced as increases in ratio of LC3 II/I and protein P62 and AMPK and mTOR expressions were significantly increased in diabetic compared to nondiabetic rats, concomitant with increased postischemic myocardial infarct size and CK-MB release but decreased Akt and eNOS activation. Diabetes was also associated with increased postischemic apoptotic cell death manifested as increases in TUNEL positive cells, cleaved-caspase-3, and ratio of Bax/Bcl-2 protein expression. NAC significantly attenuated I/RI-induced increases in oxidative stress and cardiac apoptosis, prevented postischemic autophagy formation in diabetes, and reduced postischemic myocardial infarction (all *p* < 0.05).* Conclusions*. NAC confers cardioprotection against diabetic heart I/RI primarily through inhibiting excessive autophagy which might be a major mechanism why diabetic hearts are less tolerant to I/RI.

## 1. Introduction 

Ischemic heart disease is the most serious complication of diabetes, which increases morbidity and mortality [1]. Restoring of blood flow to the ischemic heart, namely, reperfusion, is the only resolution to salvage the ischemic myocardium. However, reperfusion itself also causes additional damage to ischemic heart, which was termed ischemic reperfusion injury (I/RI). Numerous studies have demonstrated that ischemia and reperfusion induced burst production of reactive oxygen species (ROS) and the consequent oxidative damage and inflammation [2] are major mechanisms of I/RI. Increased ROS also contribute to reduced bioavailability of the cardiovascular protective nitric oxide and the impairments of the prosurvival reperfusion injury salvage kinase (RISK) pathway which involves PI3K/Akt and the survival activating factor enhancement (SAFE) pathway which involves Jak/signal transducer and activator of transcription 3 (STAT3) [3]. The impairment of these prosurvival pathways may lead to the impairment of the molecular identity of the mitochondrial permeability transition pore (mPTP) that is decisive for cardiomyocyte survival [4], which involves the regulation of cell apoptosis and autophagy via the AMPK/mTOR signaling [5]. Hyperglycemia is the major character of diabetes, which causes ROS overproduction and interferes with oxidant and antioxidant hemostasis and exacerbates myocardial I/RI in diabetes. The antioxidant N-acetylcysteine (NAC) has been shown to reduce cardiac ROS overproduction in diabetes [6] and reduce myocardial I/RI and improve postischemic heart function in diabetic rats [7, 8] through attenuating I/RI-mediated apoptotic cell death. Recent studies show that autophagy is one of the major forms of cell death in circumstances of myocardial I/RI, and autophagy is enhanced in the myocardium of diabetic subjects at certain stages of the disease, when the diabetic hearts are more vulnerable to ischemic insult. It is unknown whether or not attenuation of autophagy may represent a mechanism whereby NAC confers cardioprotection against I/RI in diabetes.

Autophagy, which destructs long-lived or aggregated proteins and damaged organelles, is a basic function of vital life to main nutrient homeostasis, energy salvage, and degradation of old, malfunctioning organelles within a cell [9]. In addition, autophagy could provide immune protection and invade pathogens. Recent studies show that autophagy may have correlation with type II programmed cell death and can initiate cell death in different circumstances and plays different roles in neurodegenerative diseases [10], cancer [11], liver diseases [12], cardiac diseases [13], metabolic syndromes [14], ageing [15], and inflammations [16]. Previous study demonstrated that autophagy was upregulated during myocardial ischemia/reperfusion (I/R) and suggested that it may be cardioprotective during ischemia, but continuous activation of autophagy during reperfusion was detrimental [17]. Adenosine 5′-monophosphate-activated protein kinase (AMPK) is a sensor of energy molecule ATP and is activated when the ratio of ATP/ADP is decreased during exercise, hypoxia, oxidative stress, glucose deprivation, and myocardial I/R [18]. AMPK is also a major regulator/activator of autophagy [19]. On the other hand, mammalian target of rapamycin (mTOR) inhibits autophagy in the heart as reported. Activation of AMPK results in decreased mTOR activity, increased autophagy, and attenuated postischemic myocardial reperfusion injury [20, 21]. Likewise, Guo et al. reported that ischemia postconditioning reduced postischemic cardiac injury through clearing autophagosome and restoring autophagy flow [22]. However, in the diabetic myocardium, the extent of cardiac autophagy has been shown to be either enhanced or reduced depending on model and duration of the disease in which the susceptibility to ischemic insult was enhanced [23, 24]. Thus, the role of autophagy in diabetic myocardial I/RI is unclear. We hypothesize that attenuation of I/RI-induced autophagy is a major mechanism by which NAC confers cardioprotection in diabetes. Therefore, this study was designed to mainly investigate the cardioprotection of NAC in diabetic rats in relation to autophagy.

## 2. Materials and Methods

### 2.1. Induction of Diabetes

Diabetes was induced by single dose (65 mg/kg) streptozotocin (STZ) (Sigma-Aldrich, St. Louis, MO) in male Sprague-Dawley rats (250 g, 6–8 weeks) injection via tail vein as we described [25]. All rats were housed in the Laboratory Animal Service Center (University of Hong Kong) and received standard care in accordance with the principles of Animal Care of the University of Hong Kong. The experimental protocols were approved by the Committee on the Use of Live Animals in Teaching and Research (CULATR).

### 2.2. Experimental Protocol

Rats were randomly divided into four groups: Ctrl: nondiabetic control; D4w: 4-week diabetes; D4w + I/R: 4 weeks' diabetic rats with ischemia/reperfusion; D4w + I/R + NAC: 4 weeks' diabetic rats treated with N-acetylcysteine (NAC) and were subjected to ischemia/reperfusion (Sigma-Aldrich, St. Louis, MO). The chemicals were dissolved in drinking water for 4 weeks' duration of treatment starting one week after induction of diabetes. We selected a dose of NAC at 1.5 g/kg/day, as our previous study has reported that NAC at the dose of 1.4–1.5 g/kg/day completely prevented hyperglycemia-induced oxidative stress after 8 weeks of STZ-induced diabetic rats [26].

I/R was achieved by occluding the left anterior descending (LAD) artery for 30 minutes followed by 2-hour reperfusion and myocardial infarction was determined using TTC (1%, 2,3,5-triphenyltetrazolium chloride) staining as described [27]. Briefly, at the end of reperfusion, the LAD was reoccluded; 5% Evans Blue was injected through the right jugular vein to mark the normal region of the left ventricle (LV). At the completion of the experiments, the rats were euthanized with overdose pentobarbital injection, and the hearts were quickly removed and cut into five 1–1.5 mm cross-sectional slices and incubated in 1% TTC in PBS at room temperature for 20 minutes. The slices were then fixed with 10% formalin overnight. TTC stained area was targeted as the region of survival area. Parts of the LV tissues were selected for Western Blot. PowerLab monitoring system (ML750 PowerLab/4sp with MLT0380 Reusable BP Transducer; AD Instruments, CO Springs, CO) was used to monitor the hemodynamics during myocardial I/R process.

### 2.3. Plasm and Cardiac Levels of Free 15-F2t-Isoprostane (15-F2t-IsoP)

Free 15-F2t-IsoP, a specific marker of oxidative stress in vivo originally produced by the random oxidation of tissue phospholipids, was measured by using an enzyme immunoassay kit (Cayman chemical, Ann Arbor, MI) as described [28]. The absorbance from the enzymatic reaction was detected at 412 nm. The values of plasma or cardiac free 15-F2t-IsoP were expressed as pg/mL in the plasma. After 2 hours of reperfusion blood samples were collected from carotid artery with anticoagulation heparin and then centrifuged to separate plasma as we described [27].

### 2.4. Plasma Biochemical Analysis

Plasma tumor necrosis factor-*α* (TNF-*α*) level was determined using rat TNF-*α* ELISA kit (eBiosource International, Burlington, Ontario, Canada). Plasma creatinine kinase-MB (CK-MB) levels were determined using a commercially available rat ELISA kit (R&D Systems, Minneapolis, MN). Plasma interleukin-6 (IL-6) level was determined using a rat IL-6 ELISA kit (eBiosource International, Burlington, Ontario, Canada). The assays were performed following the manufacturer's instructions.

### 2.5. In Situ Apoptotic Cell Death Detection

Apoptotic cell death detection was achieved using TdT-mediated DUTP-X nick end labeling (TUNEL stain) according to the manufacturer's instruction (Roche Applied Science, Indianapolis, IN, USA). First, left ventricular tissue embedded by paraffin was sliced (5 mm thick sections) and deparaffinized. Second, the sections were permeabilized using proteinase K (30 mg/mL, 30 minutes, 37°C) and were washed in phosphate buffered saline (PBS). Then as the manufacturer's instructions described to detect the apoptosis cells, DNase I was used to induce DNA strand breaks as positive control and TdT was omitted from the reaction mixture as negative control.

### 2.6. Western Blot Analysis

Proteins from frozen LV tissues were homogenized in 1x lysis buffer from Cell Signaling Technology (Beverly, MA) and centrifuged at speed of 13200 *g* for 30 minutes. The supernatant was collected as total myocardial protein. The supernatant was collected as total myocardial protein. The concentrations of protein were then determined using the Bradford protein assay.

Equal protein amounts from rat heart homogenate were resolved by 7.5–12.5% SDS-PAGE and subsequently transferred to polyvinylidene nitrocellulose membranes and processed as previously described [28]. Primary antibodies against AMPK*α*, phosphorylation AMPK*α*, Akt, phosphorylation Akt (Ser-473), LC3, P62, mTOR, phosphorylation PTEN, Bax, Bcl-2, caspase 3, cleaved-caspase-3, and GAPDH were purchased from Cell Signaling Technology (Beverly, MA). Protein bands were detected by a standard ECL method and images were measured by a densitometer.

### 2.7. Statistical Analysis

All values are expressed as means ± standard error of the mean (SEM). One-way analysis of variance (ANOVA) was used for statistical analyses (GraphPad Prism, USA) of data obtained within the same group and between groups, respectively, followed by Tukey's test for multiple comparisons of group means. *p* values less than 0.05 were considered to indicate statistically significant differences.

## 3. Results

### 3.1. The Effects of NAC on General Characters, Postischemic Myocardial Infract Size (IS), and Heart Function in Diabetic Rats

First, we observed the effect of NAC on general characters in diabetic rats. As shown in Table 1, in STZ-induced diabetic rats, plasma glucose, water intake, and food consumption were significantly increased compared to nondiabetic rats (all *p* < 0.05). After NAC treatment, food consumption and water intake were significantly reduced compared to diabetic group (all *p* < 0.05), but NAC had no significant effect on plasma glucose in diabetic rats (*p* > 0.05). Body weight in diabetic rats was significantly reduced, and NAC had no significant impact on the body weight.

**Figure 1:**
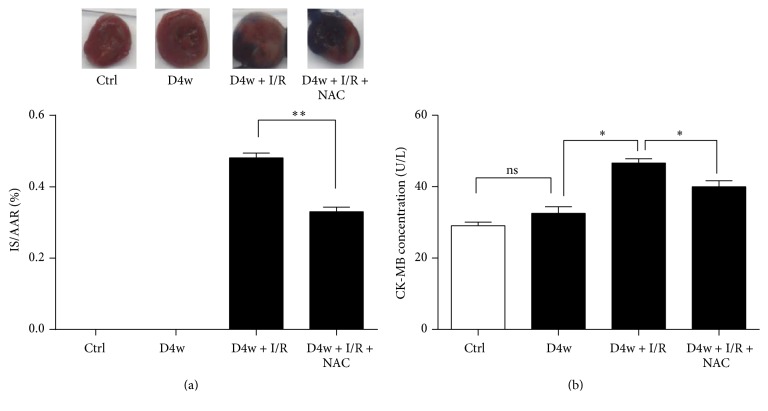
The effects of NAC on heart function and infract size (IS) in diabetic rats. (a) Infarct size (IS) is expressed as a percentage of the area at risk (AAR). (b) CK-MB release. Ischemia reperfusion (I/R) was achieved by 30-minute ischemia followed by 2-hour reperfusion in diabetic rats with or without NAC. Ctrl: nondiabetic control; D4w: 4-week diabetes; D4w + I/R: 4-week diabetic rats with ischemia/reperfusion; D4w + I/R + NAC: 4-week diabetic rats treated with N-acetylcysteine (NAC) and were subjected to ischemia/reperfusion. Dates are expressed as mean ± SEM (*n* = 6 per group). *p* < 0.05 versus D group before ischemia; ^*∗*^*p* < 0.05, ^*∗∗*^*p* < 0.01, and ns: *p* > 0.05 (no statistical significance).

As shown in Figure 1(a), NAC significantly reduced the postischemic myocardial infarct size (IS) in diabetic rats (*p* < 0.01, NAC + D4w + I/R versus D4w + I/R). And postischemic plasma CK-MB level after 2 hours' reperfusion was significantly higher compared to sham operated diabetic group (*p* < 0.05 D4w + I/R versus D4W). NAC significantly reduced postischemic CK-MB release, in accordance with lower IS (*p* < 0.05).

As shown in Table 2, baseline hemodynamics dates were similar among groups. Heart rate (HR) at baseline was not different among the 4 groups. Coronary artery occlusion (ischemia) reduced mean arterial pressure (MAP) and rate-pressure product (RPP) in all groups in comparison with baseline MAP. No significant differences in HR or RPP were observed between groups during ischemia and reperfusion. NAC treatment facilitated recovery of MAP after reperfusion as compared to the diabetic untreated group.

### 3.2. Effects of NAC on Plasma 15-F2t-Isoprostane (15-F2t-IsoP), Interleukin-6 (IL-6), and Tumor Necrosis Factor-*α* (TNF-*α*) Levels

**Figure 2:**
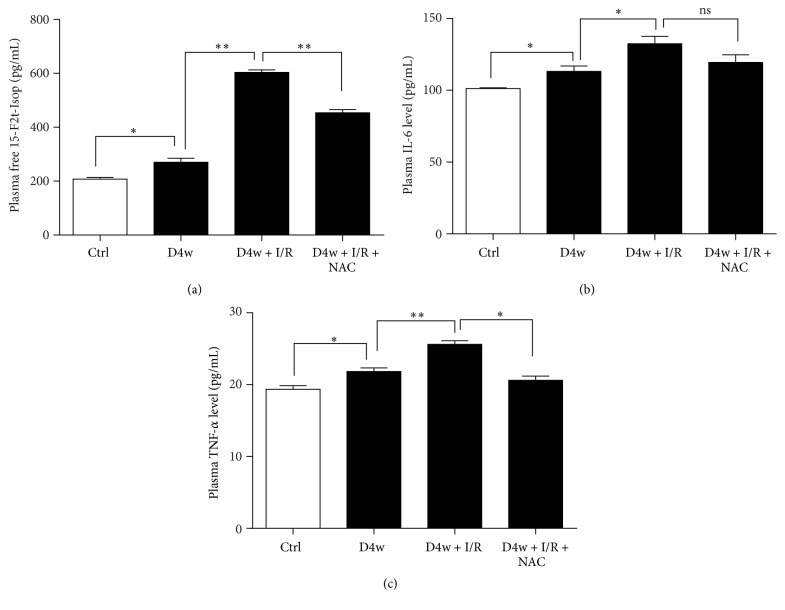
Effects of NAC on plasma 15-F2t-IsoP, IL-6, and TNF-*α* releasing. (a) Plasma level of IL-6, (b) plasma level of TNF-*α*, and (c) plasma level of 15-F2t-IsoP. Ischemia reperfusion (I/R) was achieved by 30-minute ischemia followed by 2-hour reperfusion in diabetic rats with or without NAC. Ctrl: nondiabetic control; D4w: 4-week diabetes; D4w + I/R: 4-week diabetic rats with ischemia/reperfusion; D4w + I/R + NAC: 4-week diabetic rats treated with N-acetylcysteine (NAC) and were subjected to ischemia/reperfusion. Dates are expressed as mean ± SEM (*n* = 6 per group), ^*∗*^*p* < 0.05  ^*∗∗*^*p* < 0.01.

We determined plasma 15-F2t-IsoP (specific marker of oxidative stress), IL-6, and TNF-*α* levels in control and diabetic rats with or without NAC treatment. As shown in Figures 2(a), 2(b), and 2(c), plasma IL-6 and TNF-*α* levels were increased in the rats with diabetes along with significant increase of 15-2t-IsoP (all *p* < 0.05 D4w versus nondiabetic group), and these were all further exacerbated by myocardial I/RI (*p* < 0.05, D4w + I/R versus D4w). NAC treatment significantly reduced but did not prevent the increase of plasma IL-6 level in diabetic rats. By contrast, NAC significantly decreased plasma TNF-*α* and 15-F2t-IsoP level compared to diabetic rats subjected to I/R (all *p* < 0.05).

### 3.3. Effects of NAC on Postischemic Myocardial Apoptosis, Bax/Bcl-2, and Caspase-3 Expression

**Figure 3:**
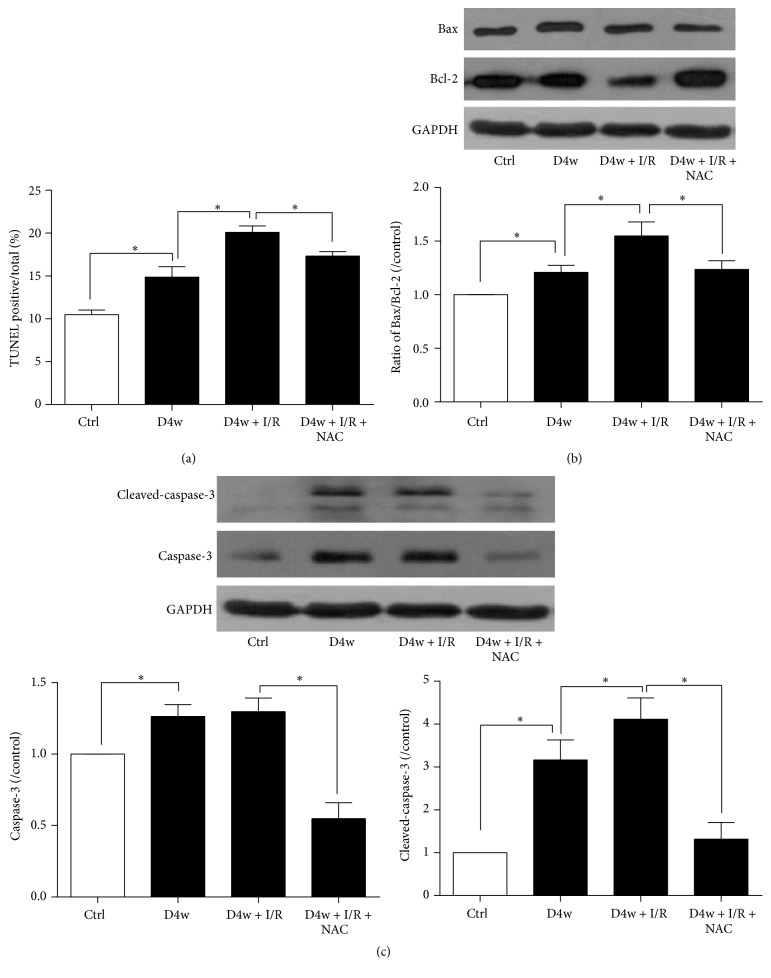
Effects of NAC on apoptosis. (a) Myocardial cell apoptosis assessed by TUNEL, (b) ratio of Bax/Bcl-2, and (c) expression of cleaved-caspase-3. Ischemia reperfusion (I/R) was achieved by 30-minute ischemia followed by 2-hour reperfusion in diabetic rats with or without NAC. Ctrl: nondiabetic control; D4w: 4-week diabetes; D4w + I/R: 4 weeks' diabetic rats with ischemia/reperfusion; D4w + I/R + NAC: 4 weeks' diabetic rats treated with N-acetylcysteine (NAC) and were subjected to ischemia/reperfusion. Dates are expressed as mean ± SEM (*n* = 6 per group), ^*∗*^*p* < 0.05.

As Figure 3(a) showed, STZ-induced diabetic rats had a significant larger number of TUNEL-staining positive cells compared to nondiabetic group (*p* < 0.05) with concomitant increases in the ratio of Bax/Bcl-2 and cleaved-caspase-3, and myocardial I/R further increased the number of apoptotic cells, while NAC treatment significantly attenuated the apoptotic cells induced by I/R (*p* < 0.05). NAC significantly attenuated the increase in the ratio of Bax/Bcl-2 and prevented diabetes and I/RI-induced increase in cleaved-caspase-3 expression (all *p* < 0.05).

### 3.4. Effects of NAC on Diabetes Induced Myocardial Autophagy and Its Related Proteins

**Figure 4:**
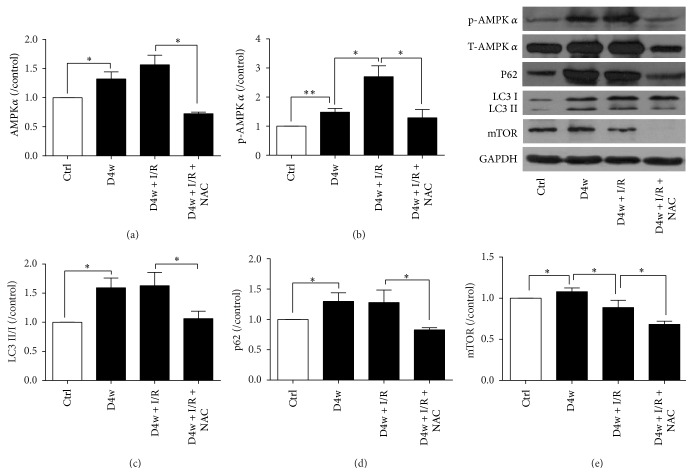
Effects of NAC on autophagy pathway. (a) Protein AMPK expression; (b) p-AMPK; (c) ratio of LC3 II/I; (d) protein P62 expression; (e) protein mTOR expression. Ischemia reperfusion (I/R) was achieved by 30-minute ischemia followed by 2-hour reperfusion in diabetic rats with or without NAC. Ctrl: nondiabetic control; D4w: 4-week diabetes; D4w + I/R: 4 weeks' diabetic rats with ischemia/reperfusion; D4w + I/R + NAC: 4 weeks' diabetic rats treated with N-acetylcysteine (NAC) and were subjected to ischemia/reperfusion. Dates are expressed as mean ± SEM (*n* = 6 per group), ^*∗*^*p* < 0.05, and ^*∗∗*^*p* < 0.01.

Autophagy has been shown to play major roles in I/RI. As Figures 4(a) and 4(b) showed, total and phosphorylated AMPK*α* (p-AMPK*α*), a strong initiator of autophagy, were significantly increased in the myocardium of diabetic rats compared to that in the nondiabetic rats (*p* < 0.05). I/R further increased p-AMPK*α* (*p* < 0.05) but did not have significant effect on AMPK*α*. The ratio of LC3 II/I represents the extent of autophagy. As Figures 4(c) and 4(d) showed, STZ-induced diabetic rats had significant higher ratio of LC3 II/I compared to nondiabetic group (*p* < 0.05). P62, which functions to clear the autophagosome in order to keep hemostasis, was also increased significantly in the myocardium of diabetic rats (*p* < 0.05, D4w versus nondiabetic ones). I/R did not further significantly increase LC3 II/I and P62. NAC treatment completely prevented diabetes induced increases of LC3 II/I and p62 proteins expression both before and after I/R as compared to D4w + IR (*p* < 0.05). In diabetic group, myocardial mTOR protein expression significantly increased (Figure 4(e)) in parallel with the significant increase in p-AMPK*α*. However, during I/R, myocardial mTOR moderately and significantly reduced in response to I/R induced further increase in p-AMPK*α* (all *p* < 0.05, D4w + I/R versus D4w). NAC treatment prevented diabetes and I/R induced increases in mTOR (*p* < 0.05, D4w + I/R + NAC versus D4w + I/R).

### 3.5. Changes of PTEN, Akt, and eNOS after NAC Treatment

**Figure 5:**
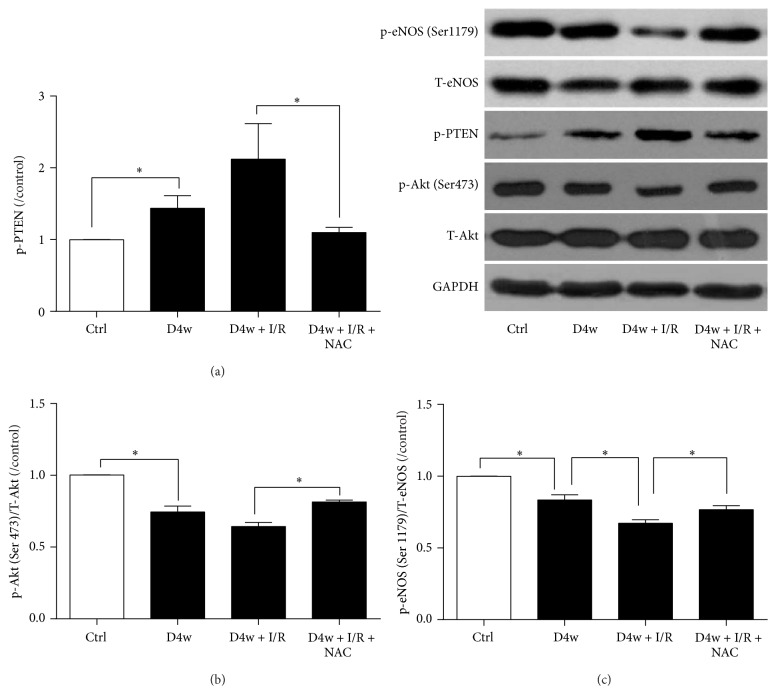
Changes of PTEN, Akt, and eNOS after NAC treatment. (a) Protein p-PTEN expression; (b) protein p-Akt expression; (c) protein p-eNOS expression. Ischemia reperfusion (I/R) was achieved by 30-minute ischemia followed by 2-hour reperfusion in diabetic rats with or without NAC. Ctrl: nondiabetic control; D4w: 4-week diabetes; D4w + I/R: 4 weeks' diabetic rats with ischemia/reperfusion; D4w + I/R + NAC: 4 weeks' diabetic rats treated with N-acetylcysteine (NAC) and were subjected to ischemia/reperfusion. Dates are expressed as mean ± SEM (*n* = 6 per group), ^*∗*^*p* < 0.05.

PTEN has been shown to play important roles in myocardial I/R; suppression of PTEN could reduce I/RI manifested as reduced infract size, increased Akt and eNOS activation, and improved heart function [29, 30]. In accordance with the previous studies, cardiac phosphorylated PTEN (p-PTEN) protein expression (Figure 5(a)) was increased significantly by hyperglycemia (*p* < 0.05, D4w versus nondiabetic rats), and I/R further increased p-PTEN expression in diabetes. Compared to nondiabetic group, cardiac phosphorylated Akt (p-Akt) and eNOS (Figures 5(b) and 5(c)) were significantly reduced in diabetic group that was concomitant with significant increase in cardiac p-PTEN. NAC treatment completely prevented diabetes and I/R induced increases of p-PTEN and significantly attenuated the reduction of p-Akt and p-eNOS induced by I/R (all *p* < 0.05, D4w + I/R + NAC versus D4w + I/R).

## 4. Discussion

Hyperglycemia was the major character of diabetes along with enhanced oxidative stress. In the current study, 15-F2t-IsoP level was significantly increased in 4 weeks' diabetic rats, and NAC treatment reduced the 15-F2t-IsoP level along with attenuating I/RI. These findings are in line with findings of recent studies by us and other groups showing that antioxidant treatment with NAC can attenuate myocardial I/RI in diabetes. The novel finding of the current study is that autophagy is increased in the myocardium of 4 weeks' diabetic rats which may be the major mechanism that rendered the diabetic heart more vulnerable to ischemic insult and that NAC treatment attenuates myocardial I/RI in diabetes primarily via inhibiting and preventing excessive autophagy.

In the heart, autophagy plays critical roles in many cardiovascular diseases in response to pathological stimuli, including cardiac hypertrophy, heart failure, and myocardial I/R. Autophagy is indicated to be promoted as a response to the ATP depletion and to the ROS accumulation [31, 32]. In diabetic mice, with autophagy-associated-gene (ATG5) knockout, autophagy was impaired, leading to mitochondria dysfunction and ROS overproduction [33], suggesting that maintaining normal function of autophagy is critical for diabetic heart functioning. In our previous study, we found antioxidant NAC treatment could reduce ROS level and I/RI in diabetic rats [8], and in the current study we found that NAC could prevent diabetes and I/R induced excessive autophagy and restore the normal autophagy function in diabetic rats subjected to I/R and subsequently reducing I/RI. The extent of cardiac autophagy has been reported to be either reduced at [34] or enhanced [35] in diabetes during different stages of the disease, but the impact of autophagy on diabetic heart vulnerability to ischemic insult is largely unknown. While moderate increase of autophagy during myocardial ischemia has been shown to be beneficial, excessive autophagy during reperfusion is harmful [24]. Mitochondria serve as the power of a cell, especially in cardiac myocytes which have a large energy requirement of energy. Mitochondrial permeability transition pore (mPTP) plays a key role in myocardial I/RI. The increase of ROS during I/RI enhances the likelihood of mPTP opening upon reperfusion [4]. In this study, excessive autophagy was found in 4 weeks' diabetic rats with myocardial I/R, which was accompanied with high levels of 15-F2t-IsoP, IL-6, TNF-*α*, and apoptosis, and NAC treatment for 4 weeks attenuated all these changes, which should have reduced mPTP opening, although further study is needed to confirm it. And, the potential causal relationship between diabetes induced excessive autophagy, apoptosis, inflammation, and oxidative stress in the context of myocardial I/RI has yet to be determined.

Autophagy is not only associated with cell survival but has also tight relationship with cell death. As reported, autophagic cell death is caspase independent and is clearly distinct from apoptotic cell death but they can occur in mixed forms with autophagic and caspase related apoptotic features. Our results showed that NAC could reduce I/R induced cell apoptosis, manifested by reducing positive apoptosis cells, the ratio of Bax/Bcl-2, and expression of cleaved-caspase-3 but has more profound effect on autophagy because it could attenuate autophagy to a greater extent. This suggests that, compared to apoptosis, autophagy played major roles in diabetic I/RI, and reducing excessive autophagy may be a potential therapy for I/RI in diabetes.

AMPK is a serine-threonine kinase which functions primarily as a metabolic sensor to coordinate anabolic and catabolic activities in the cell to maintain the cellular energy homeostasis via the phosphorylation of multiple proteins involved in metabolic pathways [36]. It has effects on many signaling pathways, such as autophagy and apoptosis [37, 38]. Our results showed that, in early diabetic rats, p-AMPK*α* was significantly increased which was induced by either diabetes or diabetes with I/R and that the increase of cardiac mTOR in diabetes was not sufficient to combat p-AMPK*α* to stimulate autophagy, and this led to excessive autophagy as reflected by significant increases in the ratio of LC3 II/I and protein P62 expression and exacerbated postischemic I/RI. Treatment with NAC completely prevented diabetes I/R induced increase in p-AMPK*α* and reduced the ratio LC3 II/I and protein P62 expression to levels comparable to normal control and concomitantly reduced mTOR. The fact that I/R in diabetes did not further increase the extent of autophagy despite I/R induced further significant increase of p-AMPK*α* and concomitant moderate but significant reduction in mTOR may suggest that excessive cardiac autophagy already occurred in diabetes is the major contributor of myocardial I/RI. The finding that NAC could reduce the activation of cardiac p-AMPK*α* is in line with our previous study [28]. In the current study, NAC restored the autophagy flow, prevented diabetes and I/R induced increases of p-PTEN, and attenuated the reduction of p-Akt and p-eNOS induced by I/R to confer cardioprotection effect in diabetic rats. Inhibition of p-PTEN has been shown to attenuate myocardial I/RI in diabetic rats [39]. However, the potential interplay between PTEN and autophagy and their regulating molecules such as mTOR in the context of diabetic myocardial I/RI has not been reported. Findings from our current study may serve to stimulate further in-depth studies in this interesting and important area to foster the development of effective therapies in combating diabetic myocardial I/RI.

In summary, we found that autophagy was excessive in 4 weeks' diabetes and this was detrimental to I/RI. Antioxidant treatment with NAC could suppress autophagy in 4 weeks' diabetic rats and reduce I/RI. NAC confers cardioprotection against diabetic heart I/RI primarily through inhibiting excessive autophagy formation following reducing oxidant stress in diabetes.

### 4.1. Clinical Perspectives

Patients with diabetes are more vulnerable to myocardial I/RI. The present study was designed to investigate the role of autophagy on NAC conferring cardioprotection effects in diabetic myocardial I/R injury. Our results showed that autophagy was excessive in 4 weeks' diabetes and this was detrimental to I/RI. Antioxidant treatment with NAC could suppress autophagy in 4 weeks' diabetic rats and reduce I/RI. It is strongly suggested that targeting inhibition of excessive autophagy may be a potential therapeutic strategy for the treatment of diabetes-associated cardiac disease in patients. Patients with acute myocardial infarction usually had increased levels of oxidative stress that were associated with a reduction in enzymatic antioxidant reserve in particular in patients with diabetes, while conditional treatments such as glucose-insulin-potassium solution did not improve these abnormalities among patients undergoing primary angioplasty [40]. Findings from our current study may suggest antioxidant treatment with NAC as a potential adjunct therapy.

## Figures and Tables

**Table 1:** General characteristics after STZ injection at termination of study.

Parameters	C	D4w	D4w + NAC
Water intake (mL/kg/day)	126.3 ± 3.2	801.2 ± 12.9^*∗*^	398.7 ± 8.3^*∗*#^
Food consumption(g/kg/day)	70.1 ± 3.8	182.7 ± 14.1^*∗*^	129.8 ± 12.7^*∗*#^
Body weight (g)	492.5 ± 14.7	326 ± 7.9^*∗*^	301 ± 11.7^*∗*^
Plasma glucose (Mm)	6.1 ± 0.2	27.9 ± 1.2^*∗*^	26.8 ± 1.3^*∗*^

All values are expressed as mean ± SEM. *n* = 6 per group, water intake and food consumption values were the average value of 4 weeks. Body weight, plasma glucose, and heart/Body weight ratio were measured at 4 weeks after STZ injection. ^*∗*^*p* < 0.05 versus control ^#^*p* < 0.05  versus D4w.

**Table 2:** Hemodynamics at baseline, at 2 hours of reperfusion in nondiabetic or diabetic rats with or without NAC treatment.

	C	D	D + I/R	D + NAC + I/R
Baseline				
HR (bpm)	268 ± 9	300 ± 9	298 ± 10	315 ± 13
MAP (mmHg)	118 ± 5	110 ± 5	113 ± 4	114 ± 3
RPP (mmHg min-1/1000)	33 ± 3	32 ± 4	34 ± 3	33 ± 3
2-hour reperfusion				
HR (bpm)	—	—	275 ± 6	300 ± 6
MAP (mmHg)	—	—	70 ± 4^*∗*^	75 ± 4^*∗*#^
RPP (mmHg min-1/1000)	—	—	22 ± 2^*∗*^	24 ± 3^*∗*^

HR: heart rate; MAP: mean arterial pressure; RPP: rate-pressure product. HR, MAP, and RPP were measured at 2 hours of reperfusion. All values are expressed as mean ± SEM (*n* = 6 per group). ^*∗*^*p* < 0.05  versus their corresponding baseline; ^#^*p* < 0.05  versus D + I/R.
